# Correlation Between Microbial Communities and Volatile Organic Compounds in Camel Milk at Different Lactation Stages in Xinjiang, China

**DOI:** 10.3390/foods15101804

**Published:** 2026-05-20

**Authors:** Qianqian Duo, Yan Zhao, Henigul Osman, Wei Shao, Yankun Zhao

**Affiliations:** 1 Xinjiang Key Laboratory of Agro-Products Quality & Safety, Laboratory of Quality & Safety Risk Assessment for Agro-Products (Urumqi), Ministry of Agriculture and Rural Affairs, Institute of Quality Standards & Testing Technology for Agro-Products, Xinjiang Academy of Agricultural Sciences, Urumqi 830091, China; duoqianqian0205@163.com (Q.D.);; 2 Xinjiang Meat and Milk Herbivore Nutrition Laboratory, College of Animal Science, Urumqi 830052, China

**Keywords:** camel milk, lactation period, volatile compounds, gas chromatography–mass spectrometry, metagenomics

## Abstract

The aroma of camel milk is a key sensory indicator for evaluating its quality and flavor. Camel milk collected at different lactation stages exhibits unique flavor characteristics. However, no systematic study has yet explored the aroma characteristics and variation patterns of camel milk across these stages. This study employs HS-SPME-GC-MS, multivariate statistical analysis, and metagenomics to systematically reveal differences in aroma formation in camel milk across lactation periods and their interactions with microbial communities. A total of 577 metabolites is detected. Through OPLS-DA screening, 24 key differential flavor compounds are identified. ROAV analysis indicates that 2,4-undecadienal and (E)-2-undecenal are the main contributors to the fatty, creamy, fresh green, and citrus aromas of camel milk. Some compounds are more abundant in colostrum, while others are richer in mature milk. For microbiota, colostrum is dominated by *Proteobacteria*, *Psychrobacter*, and *Janthinobacterium*, whereas mature milk is dominated by *Acinetobacter* and *Moraxella*. Mature milk shows significantly higher alpha diversity and species richness. Spearman correlation analysis shows that core bacterial groups such as *Enterococcus* and *Lactococcus* are significantly positively correlated with characteristic flavor compounds, including aldehydes and lactones. This finding suggests that HS-SPME-GC-MS, combined with multivariate analysis, effectively distinguishes patterns associated with microbes and flavor metabolites in camel milk at different lactation stages, which provides a theoretical basis for quality control and further processing of camel milk.

## 1. Introduction

Camel milk possesses distinct regional characteristics and profound cultural heritage. It has long been regarded by nomadic peoples as an important nutritional source. Compared with cow milk, camel milk shows higher contents of medium-chain fatty acids, lower cholesterol and lactose, and higher vitamin C [1]. It also contains higher concentrations of various bioactive components, such as lactic acid bacteria, lactoferrin, lysozyme, casein, and immunoglobulins [2,3]. It is rich in multiple nutrients essential for human metabolism, which helps regulate physiological functions, enhance immunity, and prevent disease [4]. It shows significant resource advantages in the development of specialty dairy products.

Volatile organic compounds represent core indicators that determine the sensory quality and market acceptance of camel milk. Their composition and content directly affect the flavor characteristics of dairy products. Headspace solid-phase microextraction coupled with gas chromatography–mass spectrometry (HS-SPME-GC-MS) features simple sample preparation and high sensitivity. It becomes a mainstream method for the accurate detection of volatile compounds in milk samples [5,6], which efficiently identifies key flavor substances such as aldehydes, ketones, and lactones [7]. Current studies mostly focus on flavor changes before and after camel milk processing [8], Systematic studies on flavor composition differences at different lactation stages and the screening of characteristic flavor substances remain limited, which makes it difficult to support the precise development of flavor-oriented products.

Flavor formation is affected by various factors, including raw material composition, processing technology, and storage conditions. Among these factors, dynamic changes in microbial communities are among the core factors associated with the generation of volatile flavor compounds. Complex microbial communities in raw milk generate aldehydes, ketones, esters, and other compounds through lipolysis, proteolysis, and sugar metabolism, which directly affect the sensory flavor of dairy products [9]. *Psychrotrophic bacteria* grow rapidly using unique nutrients in camel milk and secrete hydrolytic enzymes that induce protein and fat hydrolysis. This process leads to quality defects in raw milk such as discoloration, bitterness, increased gelation, acidity, and viscosity [10,11]. Therefore, microorganisms in camel milk play a decisive role in determining the characteristics of the final product. With the development of sequencing technology, high-throughput sequencing is widely used to analyze microbial communities in milk and dairy products [12]. However, this method shows certain errors in microbial identification and hardly meets the requirements of in-depth research [13]. The application of metagenomic sequencing provides an advantage in analyzing complex microbial community structures and gene functions. It becomes an effective tool for exploring metabolic mechanisms of milk-derived microorganisms and accurately identifies core functional microbial communities related to flavor metabolism [14,15]. Existing studies show that the lactation stage significantly affects the microbial composition of cow and goat milk [16]. However, the effect of lactation stage on microbial community succession in camel milk and the interaction mechanism between microorganisms and flavor compounds remain unclear. The relationship between core functional microbes and key flavor compounds has not been systematically studied.

As a major camel-breeding region in China, Xinjiang accounts for approximately 52% of the national camel population, with an annual camel milk output of 60,000 tons, which ranks first in China. This study focuses on camel milk at different lactation stages in Xinjiang. It uses HS-SPME-GC-MS combined with metagenomics to systematically identify characteristic flavor compounds and core microbial community composition in camel milk across lactation stages. It aims to comprehensively reveal intrinsic patterns underlying lactation-associated interactions between flavor and microbes in camel milk, providing theoretical and technical support for flavor regulation in camel milk.

## 2. Materials and Methods

### 2.1. Sample Collection and Processing

This study selects raw milk samples collected from a large-scale camel farm in Xinjiang as research materials. Healthy adult female Bactrian camels under natural lactation are used. A total of 12 milk samples is collected, including six samples in the colostrum group (C group, 0–3 days postpartum) and six samples in the mature milk group (N group, 3–6 months postpartum). Milk from each camel serves as an independent biological replicate and is not pooled among individuals. Sterile hand-milking is performed [16,17]. The udder and teats are cleaned with 75% alcohol and dried with sterile paper towels. Foremilk is discarded to reduce contamination. Milk samples are directly collected into pre-sterilized 200 mL containers. Sterile gloves are worn during the whole process to avoid environmental microbial interference. Sampling is conducted uniformly in the early morning before feeding to minimize the influence of dietary fluctuations on milk composition. After collection, all samples are temporarily stored in an incubator with ice packs (4 °C) and transported to the laboratory within 24 h for subsequent analysis.

### 2.2. Microbial Metagenomic Analysis

Total microbial DNA is extracted from camel milk using a Mag-Bind Soil DNA Kit (MP Biomedicals, Santa Ana, CA, USA) according to the manufacturer’s instructions. DNA concentration and purity are determined using a Qubit 4 Fluorometer (Invitrogen, Carlsbad, CA, USA). DNA integrity is verified by 1% agarose gel electrophoresis [18]. A sequencing library is constructed following the Illumina TruSeq DNA Sample Preparation Guide. Metagenomic sequencing is performed on the Illumina platform, which generates 10 GB of clean data per sample.

### 2.3. Solid-Phase Microextraction Extraction

Samples are removed from a −80 °C freezer and ground in liquid nitrogen, followed by vortex mixing. Approximately 0.2 mL of each sample is weighed into a headspace vial, and 0.2 g of NaCl is added to inhibit enzyme activity [19]. Then, 20 μL of 10 μg/mL 3-hexanone is added as an internal standard. The vial is incubated at 60 °C for 5 min. Headspace extraction is carried out at 60 °C for 15 min using a 120 μm DVB/CWR/PDMS SPME Arrow. The fiber is desorbed at 250 °C for 5 min and conditioned at 250 °C for 5 min before reuse.

### 2.4. GC-MS

Volatile organic compounds (VOCs) are analyzed using an Agilent 8890 GC system coupled with a 7000D triple quadrupole mass spectrometer (Agilent Technologies, Santa Clara, CA, USA). Separation is performed on a DB-5MS capillary column (30 m × 0.25 mm × 0.25 μm). Helium is used as the carrier gas at a constant flow rate of 1.2 mL/min. The injector temperature is 250 °C with a solvent delay of 3.5 min. The oven temperature program was set as follows: 40 °C for 3.5 min, 10 °C/min to 100 °C, 7 °C/min to 180 °C, 25 °C/min to 280 °C, and hold for 5 min [20]. Electron impact ionization (EI) is performed at 70 eV. The ion source, quadrupole, and transfer line temperatures are 230 °C, 150 °C, and 280 °C, respectively. Selected-ion monitoring (SIM) is used for quantification.

### 2.5. Qualitative and Quantitative Analysis of GC-MS

Qualitative and quantitative analyses of volatile compounds are conducted by comparing mass spectra with the NIST2020 library. Compounds with a similarity score >80% are selected. Retention indices (RI) are calculated using a normal alkane mixture (C7–C40) as a standard reference. Volatile compounds are finally identified by matching calculated RI values with those reported in the literature or databases [21]. In the separation and identification of volatile flavor compounds in camel milk, 20 μL of 3-hexanone (10 μg/mL) is added as an internal standard (IS). The content of each volatile flavor compound relative to 3-hexanone is calculated using the corresponding formula [22].

(1) $$X_{i}=\frac{V_{s}\times C_{s}}{V}\times \frac{I_{i}}{I_{i}}\times 10^{-3}$$

where X_i_ is the amount of compound i in the sample to be tested (μg/mL); V_s_ is the volume of internal standard added (μL); C_s_ is the concentration of internal standard (μg/mL); V is the volume of the sample to be tested (mL); I_s_ is the peak area of the internal standard; I_i_ is the peak area of compound i in the sample to be tested.

### 2.6. Relative Odor Activity Value

The relative odor activity value (ROAV) is usually applied to flavor compounds with low thresholds and high contents. A ROAV value ≥ 1 indicates that the compound directly contributes to the flavor of the sample [23].

(2) $$ROAV_{i}=\frac{C_{i}}{C_{\max}}\times \frac{T_{\max}}{T_{i}}\times 100$$

Among them: C_i_ is the relative content of compound i, %; T_i_ is the threshold of compound i, μg/L; C_max_ is the relative content of the component with the greatest overall flavor in the sample, %; T_max_ is the odor threshold of the component with the greatest overall flavor in the sample, μg/L.

### 2.7. Statistical Analysis

In GC-MS analysis, each biological replicate undergoes three technical replicates, and the average peak area is used for calculation. Data are presented as mean ± standard deviation (SD). Statistical analysis is performed using Microsoft Office Excel 2016. Graphs are generated using GraphPad Prism (v.9). Comparisons between two groups are conducted using an independent samples *t*-test, with a significance level set at *p* < 0.05. Bar charts of microbial relative abundance are generated using R (v.4.4.3). Spearman correlation analysis (|r| ≥ 0.7 and *p* < 0.05) is performed in R to evaluate the correlation between genus-level microbes and volatile flavor compounds. Random forest analysis is conducted in R to identify key microbial biomarkers with strong discrimination between groups. The top 20 genera with the highest importance scores are selected as microbial biomarkers. Multivariate analysis is performed using SIMCA 14.1 for OPLS-DA. Differential metabolites are screened using the criteria of VIP ≥ 1.2, |Log_2_FC| > 2, and FDR < 0.05 (Benjamini–Hochberg correction).

## 3. Results and Discussion

### 3.1. Analysis of Characteristic Flavor of Camel Milk at Different Stages of Lactation by GC-MS

**Figure 1:**
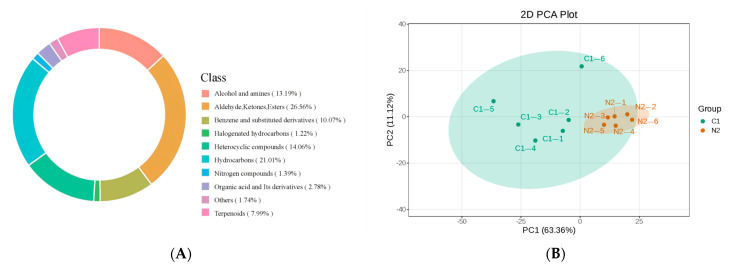
Metabolomic profiling based on GC-MS. Pie chart of metabolite class composition (**A**). Principal component analysis plot (**B**).

This study investigates aroma characteristics of camel milk at different lactation periods. HS-SPME-GC-MS is used to analyze and identify the composition and relative content of aroma compounds in camel milk. A total of 577 metabolites are detected, including 76 alcohols and amines, 154 aldehydes, ketones, and esters, 16 acids, 8 nitrogen-containing compounds, 121 hydrocarbons, 46 terpenes, 58 benzenes and their derivatives, 81 heterocyclic compounds, 7 halogenated hydrocarbons, and 10 other compounds (Figure 1A). To further explore intergroup differences, PCA analysis is performed on the C group and N group samples (Figure 1B). The result shows that PC1 explains 63.36% of the total variance, and PC2 explains 11.12% of the total variance, with a cumulative contribution rate of 74.48%, which indicates that the principal components effectively capture the main characteristics of the samples. The PCA score plot shows that the C group and N group sample points are clearly separated along the PC1 axis, and samples within each group are tightly clustered, indicating significant differences in the overall composition of flavor substances between the two groups and consistent replicates within each group.

### 3.2. OPLS-DA Analysis of Volatile Flavor Compounds

**Figure 2:**
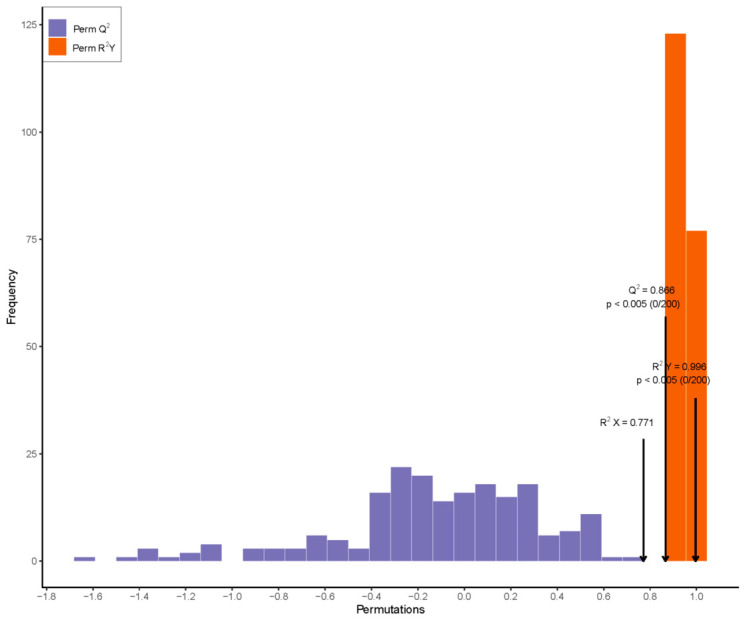
OPLS-DA plot of camel milk at different lactation stages.

The OPLS-DA model is a multivariate statistical method that models multiple independent variables and accurately identifies key variables contributing to group differences [24]. It is further used to screen key volatile compounds. The OPLS-DA model achieves complete separation of camel milk samples from different lactation stages (Figure 2). The model shows strong predictive reliability, with an independent variable fitting index (R^2^ X) of 0.771, a dependent variable fitting index (R^2^ Y) of 0.996, and a model predictive index (Q^2^) of 0.866, which confirms the accuracy of the model. R^2^ and Q^2^ values above 0.5 indicate acceptable model fitting results [25]. A 200-time permutation test is conducted, and the result shows that the original model’s R^2^Y and Q^2^ are significantly higher than those of random permutation (permutation test *p* < 0.05), which validates the model and suggests that the result can be used for discriminative analysis of camel milk samples from different lactation periods.

The result indicates that differences in volatile components between group C and group N are related to physiological changes at different lactation stages. Previous studies confirm the existence of the rumen microbiota–mammary gland regulatory axis in ruminants, which participates in regulating milk components and flavor formation [26]. Metabolic products derived from the rumen, such as acetic acid and butyric acid, are absorbed into the bloodstream and transported to the mammary gland, which provides important precursor substances for the de novo synthesis of mammary fatty acids and the generation of flavor compounds [27]. Therefore, differences in volatile components between group C and group N in this study are partly attributed to changes in rumen fermentation patterns and metabolite transport characteristics at different lactation stages. The lactation stage is a key factor related to the milk microbiome and flavor characteristics of ruminants [28]. Meanwhile, seasonal changes and variations in feed composition are also reported to be associated with changes in milk composition, which reasonably explains the observed stage differences in volatile substances in camel milk in this study [29].

Using Variable Importance in Projection (VIP) ≥ 1.0 and Log_2_ fold change (Log_2_FC) ≥ 1 as preliminary screening thresholds, a total of 193 differential flavor compounds are identified. To reduce false positives caused by multiple comparisons, the Benjamini–Hochberg method is further used to perform False Discovery Rate (FDR) correction on *p*-values. With (VIP ≥ 1.2, |Log_2_FC| > 2) and FDR < 0.05 as strict thresholds, 24 key differential flavor compounds are finally determined for subsequent ROAV analysis (Table 1).

### 3.3. ROAV Analysis of Key Volatile Compounds in Camel Milk at Different Lactation Stages

Many VFCs are detected in camel milk at different lactation stages, but only some contribute to the overall flavor of camel milk. The remaining compounds play a modifying and synergistic role in the overall flavor of camel milk at different lactation stages. Their aromatic formation is relatively complex, and the concentration of volatile compounds alone is insufficient to determine their significant contribution to the overall aroma. Therefore, the ROAV method is applied to calculate specific VFCs that affect the overall aroma of samples and to analyze the contribution of volatile flavor substances in camel milk at different lactation stages to camel milk flavor. ROAV is a common index ranging from 0 to 100. A higher ROAV value indicates a greater contribution of the flavor substance to the overall flavor of the sample [30,31]. Some volatiles with ROAV < 1 show synergistic effects with compounds of homologous structure and aroma, which may also affect the main aroma of camel milk at different lactation stages.

The result shows that a total of 13 key volatile compounds with ROAV > 0.1 are identified based on key volatile compounds (Table 2). 2,4-Undecadienal shows the highest content in colostrum with an extremely low threshold. Its value is 100 in group C and 27.72 in group N, which are much higher than those of other key flavor substances. This result indicates that this compound produces significant flavor effects and is the main contributor to green, fatty, and creamy flavor characteristics. (E)-2-Undecenal ranks second, with a value of 7.95 in group C and 1.62 in group N, which provides fresh peel and citrus aroma to samples and plays an important role in modifying the overall flavor profile. In contrast, compounds such as undecenal, benzofuranone derivatives, 2-methylnaphthalene, undecyl ketone, biphenyl, and δ-lactone show values significantly lower than 1 in both colostrum and mature milk, indicating that their direct contribution to the main flavor is limited and that they primarily serve as background harmonizers or synergistic enhancers. Among them, δ-lactone has a relatively high threshold (2.6 μg/mL), but its unique coconut and creamy aroma still produces sensory effects at low concentrations.

Trace compounds detected in this study, such as betazole, biphenyl, and 1,3-cyclohexanediamine, are common low-abundance background components in ruminant milk. These substances are believed to primarily originate from secondary metabolites of forage plants, environmental atmospheric components, and metabolic by-products of rumen fermentation microbes [32]. The detected concentrations of these compounds are extremely low. They exist only as background components in milk, do not dominate the overall flavor, and are not derived from external contamination [33]. Similar low-abundance nitrogen-containing and aromatic compounds are detected in camel milk from different regions, which are associated with environment, season, and feeding practices.

Aldehydes, ketones, and terpenes are the main classes of compounds closely related to the aroma characteristics of camel milk. 2,4-Undecadienal and (E)-2-Undecenal, as high ROAV aldehydes, are significantly enriched in group C, which may be attributed to enhanced milk fat oxidation metabolism in the early lactation period. The active release of a large amount of short-chain aldehydes through the fatty acid β-oxidation pathway aligns with the patterns of milk fat metabolism in ruminants [34].

### 3.4. Bacterial Composition and Taxonomic Annotation of Camel Milk from Different Lactations

**Figure 3:**
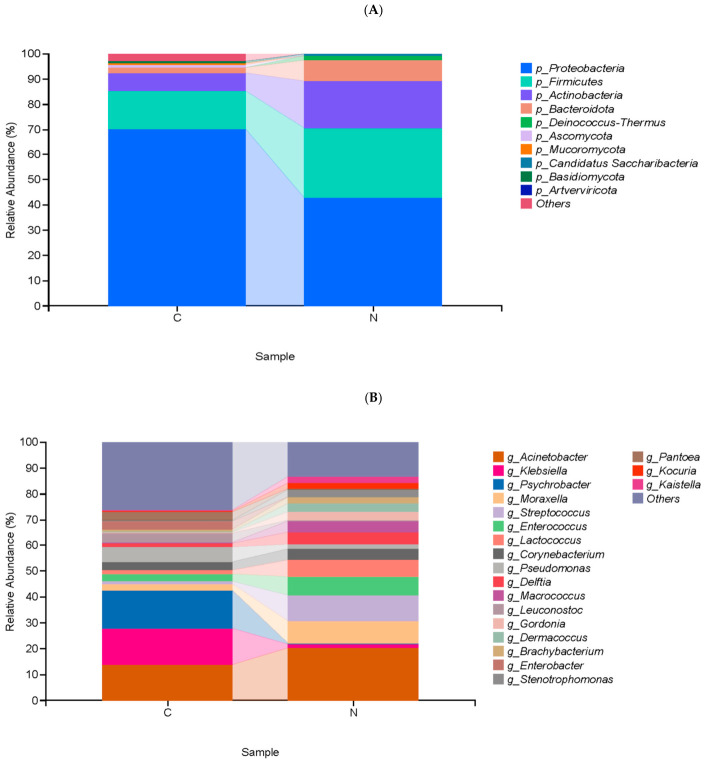

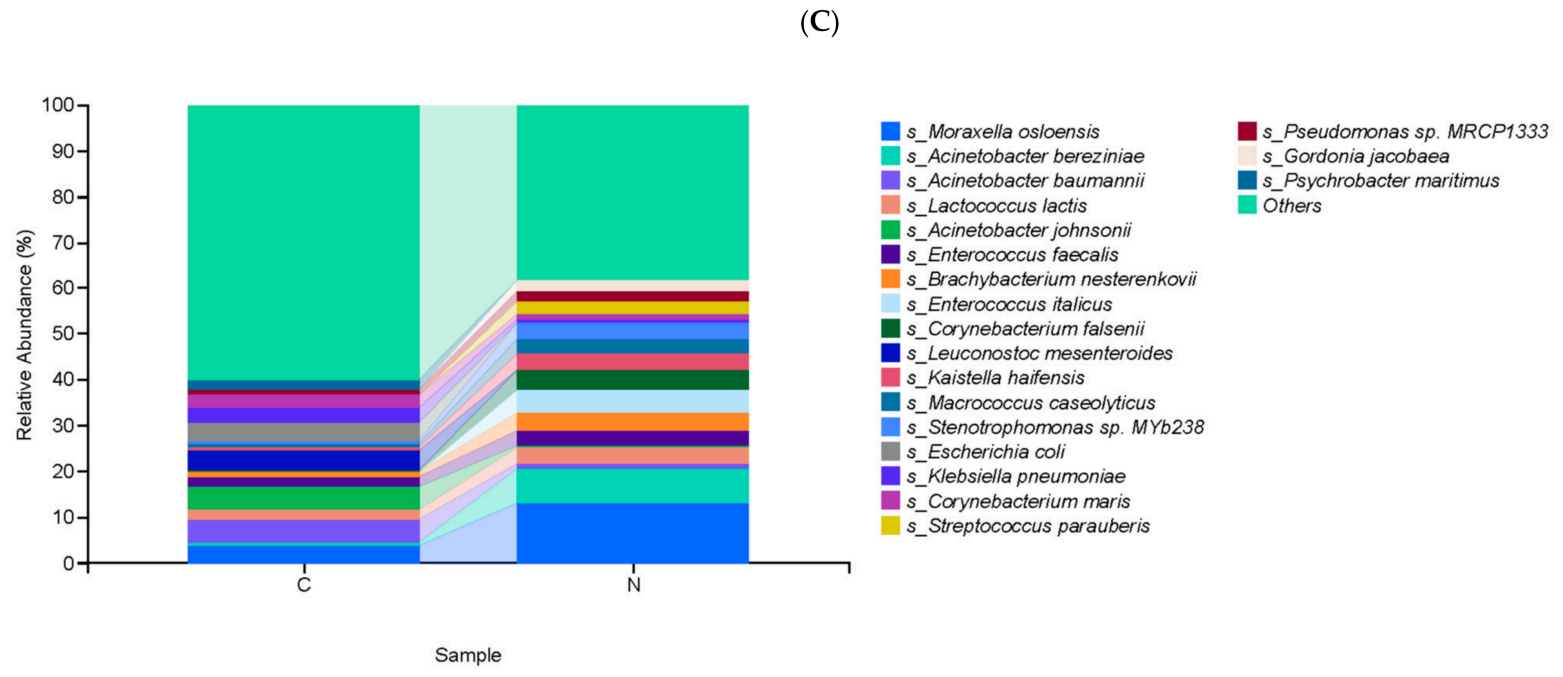
Relative abundance of (**A**) operational taxonomic units in camel milk at different lactation stages. Relative abundance of operational taxonomic units at the (**B**) genus and (**C**) species levels in camel milk at different lactation stages (colostrum, normal milk). C—colostrum; N—normal milk.

This study evaluates the composition and diversity of bacterial species in camel milk at different lactation stages. Each sample is classified according to six taxonomic levels, including phylum, genus, and species [35]. A total of 339 bacterial genera and 825 bacterial species are identified in 12 camel milk samples. At the phylum level, both groups are dominated by *Proteobacteria* (70.37% and 42.77%), *Firmicutes* (14.84% and 27.68%), *Actinobacteria* (7.15% and 18.63%), and *Bacteroidetes* (2.20% and 8.32%) (Figure 3A).

At the genus level, the composition and structure of bacterial communities across different lactation periods are similar, but the proportions of each genus vary widely. In group C, *Psychrobacter* is the most abundant genus, while in group N, *Acinetobacter* is the most abundant genus, accounting for 14.78% and 20.16%, respectively. In addition, *Pantoea* is present in group C but not detected in group N; *Kaistella* is present in group N but not detected in group C (Figure 3B). At the species level, *Acinetobacter johnsonii* is the most abundant species in group C, and *Moraxella osloensis* is the most abundant species in group N, accounting for 5.05% and 13.00%, respectively. Notably, *Escherichia coli* and *Marinobacterium psychrophilum* are only found in group C samples, accounting for 3.97% and 2.12% respectively. *Enterococcus italicus*, *Streptococcus parauberis*, and *Gordonia jacobaea* are only found in group N samples, accounting for 4.92%, 3.10%, and 2.46%, respectively (Figure 3C). Some microorganisms are found only in a single group, suggesting that the lactation stage is associated with changes in microbial community structure.

The presence of *Escherichia coli* in group C is related to immune activation in the early lactation period. High concentrations of lactoferrin and lysozyme in colostrum effectively inhibit the growth of most pathogenic bacteria [36]. The dependence of *psychrophilic* marine bacteria on low-temperature metabolic pathways enables adaptation to the relatively low mammary gland temperature during early colostrum secretion [37]. The increased abundance of *Enterococcus italicus* and *Streptococcus parauberis* is related to their lactose-metabolism advantage. Mature milk contains higher lactose content than colostrum, while *Enterococcus* efficiently transports lactose via the phosphotransferase system (PTS), and *Streptococcus parauberis* encodes β-galactosidase (lacZ) to break down lactose, which allows occupation of a nutritional niche [38,39,40]. *Gordonia jacobaea*, as a member of the *Actinobacteria phylum*, potentially participates in lipid metabolism of mature milk through its lipase and protease secretion abilities [41]. It is speculated that the composition and distribution differences of these bacterial communities are related to the physiological and chemical characteristics of camel milk at different lactation stages.

### 3.5. Alpha Diversity and Beta Diversity Analysis of Microbiota in Camel Milk from Different Lactations

**Figure 4:**
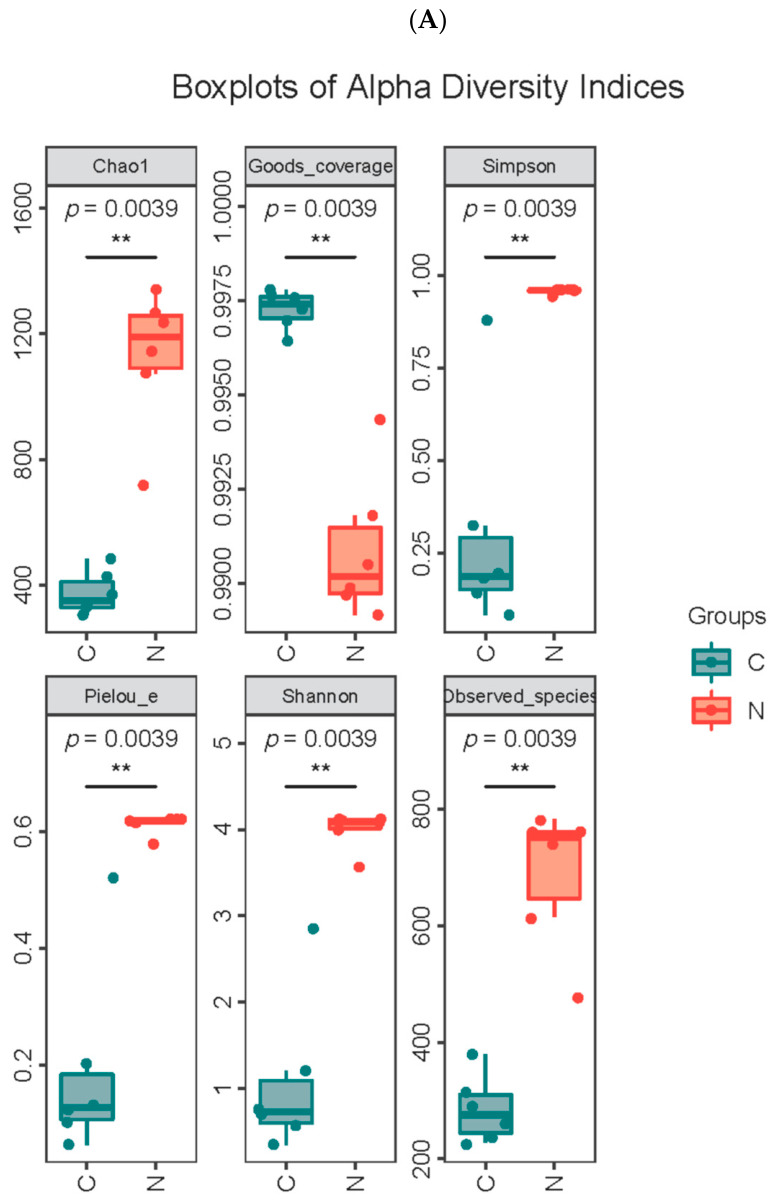

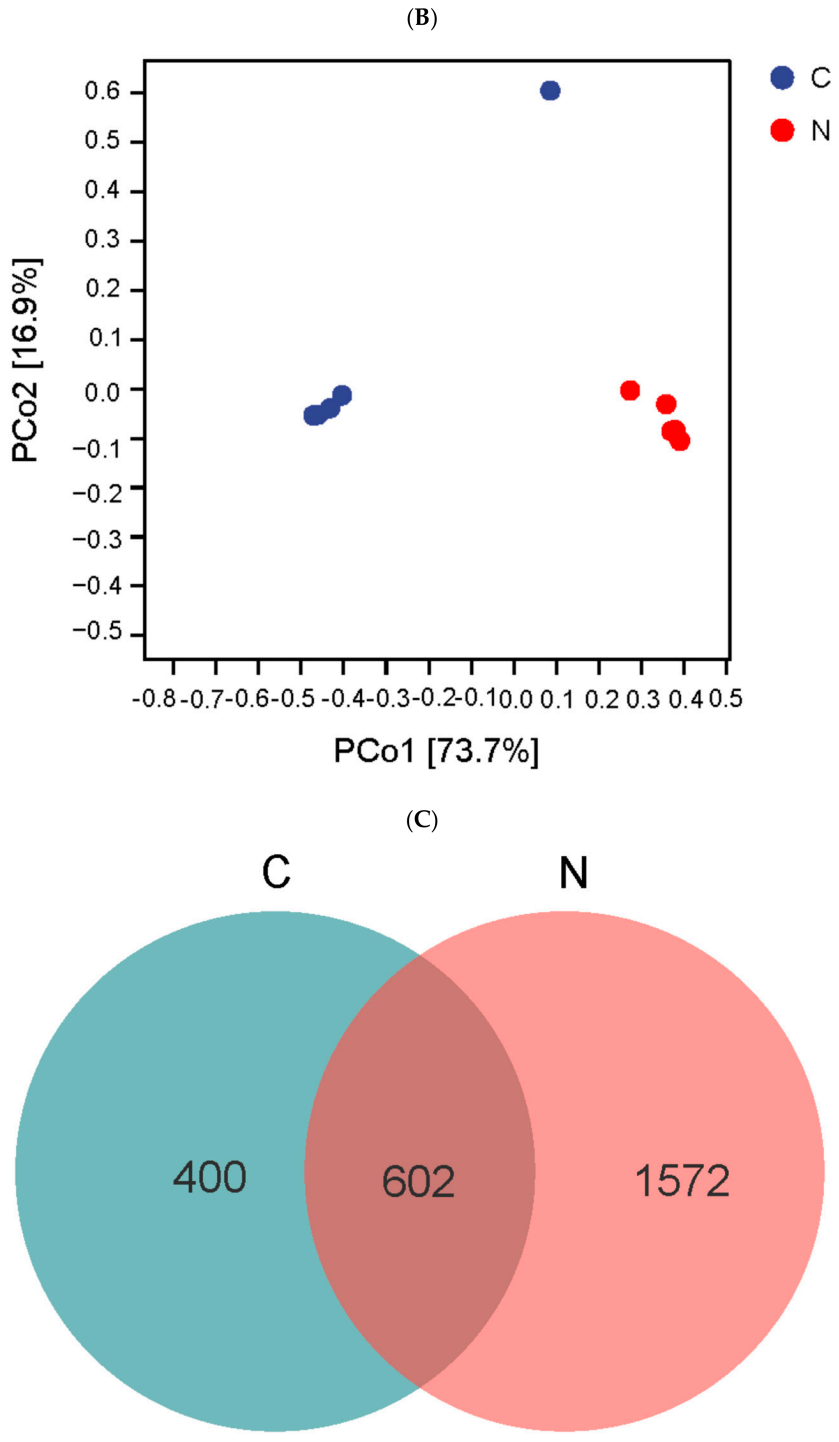
Study on the microbial diversity of camel milk at different lactation periods. (**A**) α diversity index. (**B**) β diversity analysis. (**C**) Venn diagram of taxonomic units at different lactation periods. C—colostrum; N—regular milk. ** indicate *p* < 0.01 (Wilcoxon rank-sum test).

This study analyzes microbial diversity to explore differences in microbial communities in camel milk at different lactation periods. Alpha diversity indices are comprehensive indicators that measure species richness and evenness within a sample. Chao1 and observed species reflect the richness of bacterial communities, Shannon and Simpson reflect the diversity of bacterial communities, Pielou’s evenness reflects the evenness of species within bacterial communities, and Good’s coverage reflects the extent to which the sample covers the bacterial community. The coverage rate of each sample is >99%, which indicates that the sequencing depth basically covers all bacteria and that the sequencing result reflects the real situation of microorganisms in each sample (Table 3). The richness and diversity of bacterial communities are higher in group N samples than in group C samples. The Pielou evenness index shows that the evenness of bacterial community distribution is higher in group N samples than in group C samples (*p* = 0.0039). The result shows that the bacterial Alpha diversity indices of camel milk samples in group N are generally higher than those in group C (Figure 4A and Table 3). Based on Beta diversity analysis, it is found that camel milk at different lactation periods explains 90.6% of bacterial variation (Figure 4B).

In summary, camel milk at different lactation stages shows a strong impact on the estimation of microbial alpha and beta diversity. Group N shows higher microbial diversity. This phenomenon is related to changes in mammary metabolism and environmental interactions. In the late lactation stage, lipid metabolism activity of camel mammary epithelial cells decreases, which leads to a reduction in fatty acid oxidation products in milk (such as short-chain aldehydes and ketones), thus providing niches for the colonization of more heterotrophic microbes [42]. Existing studies show that ruminant feed and seasonal changes affect rumen fermentation and microbial communities in milk [43]. The microbial community differences observed in this study are also related to changes in feed and physiology during lactation.

A Venn diagram is used to assess shared and unique microbes in camel milk at different lactation stages for better visualization of microbial community overlap. Shared bacterial community members of camel milk samples at different lactation stages account for only 602, with 1002 bacterial community members in group C samples and 2174 in group N samples. Group N samples show more bacterial community members (1572) than group C samples, which indicates higher bacterial richness of camel milk in group N samples (Figure 4C).

The random forest model further screens 20 genus-level biomarkers with significant discriminative power. The top 20 biomarkers are mainly distributed in the genera *Enterococcus* (six species), *Lactobacillus* (five species), and *Streptococcus* (four species). Among them, *Enterococcus durans* shows the highest feature importance, followed by *Lactobacillus salivarius*. In addition, some genera include both metabolic functions and potential pathogenicity, which suggests that changes in the microbial community structure affect the functional characteristics and safety of dairy products. It is noteworthy that some biomarker genera show antibacterial activity, and fluctuations in their abundance are related to mammary defense mechanisms [44].

### 3.6. Correlation Analysis Between Microorganisms and Flavor in Camel Milk at Different Lactation Stages

**Figure 5:**
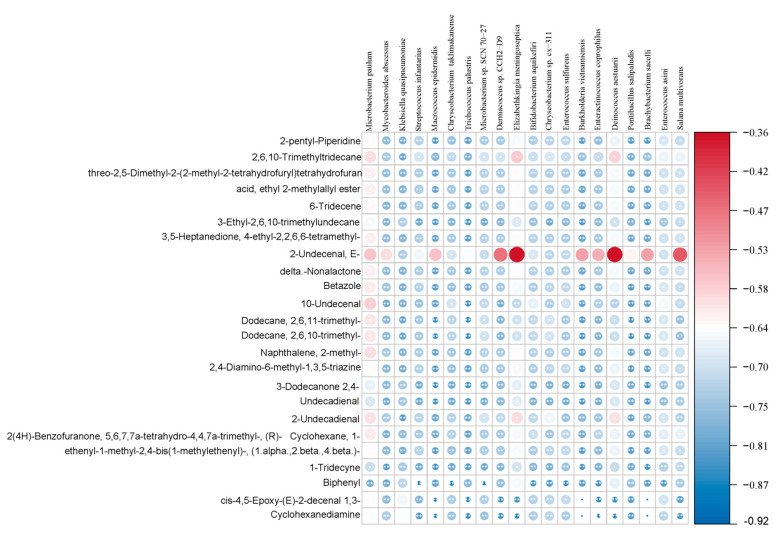
Correlation Analysis Between Key Flavor Substances and Genus-Level Microbial Relative Abundance. Note: Different colors indicate the positive or negative correlation of coefficients. The darker the color, the stronger the correlation. * indicates a significant correlation (*p* < 0.05), ** indicates a highly significant correlation (*p* < 0.01).

This study further analyzes the correlation between flavor changes in camel milk at different lactation stages and microorganisms. Spearman correlation analysis is used to explore potential associations between microorganisms and flavor substances. The interactions between microbial communities and metabolites in camel milk exhibit significant division of labor and cooperative characteristics. Spearman correlation analysis of key flavor substances with the relative abundance of genus-level microbes (Figure 5) shows that *Enterococcus durans*, *Lactococcus lactis*, unclassified *Enterococcus*, and unclassified *Lactococcus* are significantly positively correlated (*p* < 0.05) with characteristic flavor substances such as E-2-undecenal, 10-undecenal, and δ-nonanolactone, among which the correlation with E-2-undecenal is the strongest. This result suggests that these microorganisms are potential contributors to the formation of characteristic fatty and milky aromas in camel milk. From a metabolic mechanism perspective, these microorganisms are related to lipolysis and fatty acid oxidation processes, which potentially contribute to the formation of volatile flavor compounds such as aldehydes and lactones, thus conferring the unique milky and fatty aroma of camel milk [45,46]. *Lactic acid bacteria* produce diacetyl and acetoin with a buttery aroma through the citric acid metabolic pathway under the catalysis of acetolactate synthase and α-acetolactate decarboxylase [47], although these substances are not the dominant flavor components in this study. This study reports a strong correlation between *lactic acid bacteria* and (E)-2-undecenal and δ-nonanolactone, which suggests that lipolysis of medium-chain triglycerides mediated by the camel milk microbiome and subsequent ω-7 oxidation and lactonization reactions are highly active during the early lactation period [48]. Rapid quantitative methods targeting these microbial populations can be developed as flavor-quality indicators to help processing companies grade milk sources according to expected flavor characteristics.

*Enterococcus faecium*, *Lactococcus cremoris*, and *Enterococcus hirae* are moderately positively correlated with some ester and acid flavor compounds (*p* < 0.05), while *Enterococcus faecalis* and *Enterococcus casseliflavus* are significantly negatively correlated with alkane and ketone compounds (*p* < 0.05), which indicates that these microorganisms inhibit the accumulation of non-enzymatic oxidation products and reduce off-flavor formation [49]. For long shelf-life products, maintenance of a balanced microbial ecology or addition of protective cultures containing these strains reduces the formation of stale or metallic tastes [50]. Conversely, cold chain management should be improved to minimize the presence of psychotropic bacteria to prevent spoilage caused by lipolysis and proteolysis [51]. Other *Enterococcus* species show weak or no significant correlation with most flavor compounds, which indicates that their contribution to flavor is relatively low.

## 4. Conclusions

This study employs HS-SPME-GC-MS to systematically characterize the volatile flavor compounds in camel milk across different lactation stages and combines metagenomic approaches to explore the association between microbial communities and flavor compounds. A total of 577 VFCs are identified, and significant differences in the composition of flavor substances between colostrum and mature milk are observed. OPLS-DA analysis confirms a clear distinction between samples from the two stages. Based on ROAV analysis, 2,4-undecadienal and (E)-2-undecenal are identified as key aroma-active compounds. Random forest analysis selects 20 genus-level biomarkers, among which *Enterococcus* and *Lactococcus* show a significant positive correlation with characteristic aldehydes and lactones, which suggests their potential involvement in camel milk flavor formation through lipolysis and fatty acid oxidation pathways. The result of this study provides a scientific basis for camel milk flavor research and raw material grading, which lays a foundation for optimizing processing techniques and developing targeted flavor products. In the future, functional experiments are needed to verify the causal relationship between microorganisms and flavor substances and to further investigate the impact of feeding and storage conditions on flavor stability for guiding industrial applications.

## Figures and Tables

**Table 1:** Relative content of key flavor substances in camel colostrum and regular milk.

Serial Number	Material Name	Camel Colostrum (%)	Regular Camel Milk (%)	*p* Value	VIP Value
1	.delta.-Nonalactone	5.27 ± 1.91	6.72 ± 2.94	0.004	1.210
2	Betazole	4.24 ± 1.87	4.82 ± 2.27	0.008	1.204
3	1-Tridecyne	0.51 ± 0.18	0.65 ± 0.23	0.004	1.239
4	Dodecane, 2,6,11-trimethyl-	1.84 ± 0.69	2.07 ± 0.77	0.004	1.239
5	2,4-Diamino-6-methyl-1,3,5-triazine	1.51 ± 0.66	1.59 ± 0.75	0.007	1.204
6	3,5-Heptanedione, 4-ethyl-2,2,6,6-tetramethyl-	15.20 ± 6.54	14.83 ± 7.48	0.006	1.216
7	2-pentyl-Piperidine	28.83 ± 11.07	2.15 ± 14.34	0.004	1.225
8	3-Dodecanone	1.05 ± 0.42	1.25 ± 0.40	0.006	1.283
9	3-Ethyl-2,6,10-trimethylundecane	17.41 ± 6.80	19.42 ± 4.71	0.005	1.279
10	6-Tridecene	19.77 ± 7.62	21.85 ± 9.80	0.004	1.223
11	Cyclohexane, 1-ethenyl-1-methyl-2,4-bis(1-methylethenyl)-, (1.alpha.,2.beta.,4.beta.)-	0.88 ± 0.38	0.90 ± 0.41	0.006	1.239
12	2,4-Undecadienal	1.03 ± 0.32	1.28 ± 0.41	0.002	1.299
13	2,6,10-Trimethyltridecane	28.11 ± 13.87	19.37 ± 10.42	0.009	1.204
14	2-Undecenal, E-	6.41 ± 2.96	5.76 ± 3.79	0.002	1.236
15	threo-2,5-Dimethyl-2- (2-methyl-2-tetrahydrofuryl) tetrahydrofuran	25.40 ± 8.94	30.82 ± 12.62	0.003	1.228
16	Naphthalene, 2-methyl-	1.67 ± 0.65	1.86 ± 0.86	0.004	1.227
17	2(4H)-Benzofuranone, 5,6,7,7a-tetrahydro-4,4,7a-trimethyl-, (R)-	0.96 ± 0.50	0.88 ± 0.35	0.013	1.223
18	Fumaric acid, ethyl 2-methylallyl ester	20.22 ± 7.07	22.90 ± 11.33	0.003	1.240
19	10-Undecenal	1.94 ± 0.68	2.35 ± 0.86	0.003	1.219
20	2-Undecanone	0.92 ± 0.31	1.18 ± 0.50	0.002	1.224
21	Biphenyl	0.37 ± 0.14	0.25 ± 0.21	0.002	1.214
22	cis-4,5-Epoxy-(E)-2-decenal	0.27 ± 0.06	0.20 ± 0.20	0.000	1.318
23	1,3-Cyclohexanediamine	0.12 ± 0.02	0.07 ± 0.00	0.000	1.417
24	Dodecane, 2,6,10-trimethyl-	1.84 ± 0.69	2.07 ± 0.77	0.004	1.239

Note: Significant difference (*p* < 0.05); extremely significant difference (*p* < 0.001).

**Table 2:** ROAV values of key flavor substances in camel colostrum and regular milk.

Serial Number	Material Name	Flavor Description	Threshold (μg/kg)	Camel Colostrum	Regular Camel Milk
1	2,4-Undecadienal	green, buttery, spicy, baked, fruity, fatty, aldehydic, chicken	0.00001	100	27.72
2	2-Undecenal, E-	fresh, fruity, citrus, orange, peel	0.00078	7.95	1.62
3	10-Undecenal	waxy, aldehydic, rose, mandarin, citrus, soapy, fatty	0.0035	0.54	0.15
4	2(4H)-Benzofuranone,5,6,7,7a-tetrahydro-4,4,7a-trimethyl-, (R)-	musky, coumarin	0.0021	0.44	0.09
5	Naphthalene, 2-methyl-	sweet, floral, woody	0.004	0.42	0.10
6	2-Undecanone	waxy, fruity, creamy, fatty, orris, floral	0.0062	0.16	0.04
7	3-Dodecanone	-	0.0083	0.12	0.03
8	Biphenyl	pungent, rose, green, geranium	0.0033	0.11	0.02
9	.delta.-Nonalactone	coconut, creamy, sweet, milky, coumarin	2.6	0.002	0.0006

**Table 3:** Alpha diversity index of different groups of camel milk samples.

Group	Chao 1	Observed Species	Shannon	Simpson	Pielou’s Evenness	Good’s Coverage
C	372.28 ± 68.66	285.67 ± 56.56	1.07 ± 0.92	0.30 ± 0.29	0.19 ± 0.17	1 ± 0
N	1129.23 ± 223.27	689.0 ± 121.69	4 ± 0.22	0.96 ± 0.01	0.61 ± 0.02	0.99 ± 0

## Data Availability

The original contributions presented in the study are included in the article. Further inquiries can be directed to the corresponding author.
